# Recurrent acute coronary syndrome caused by refractory vasospastic angina in a patient with essential thrombocythemia: a case report

**DOI:** 10.1093/ehjcr/ytaf505

**Published:** 2025-10-04

**Authors:** Masayoshi Fujii, Noritoshi Hiranuma, Yoshitaka Ohashi

**Affiliations:** Department of Cardiology, Ako City Hospital, 1090 Nakahiro, Ako, Hyogo 678-0232, Japan; Department of Cardiology, Ako City Hospital, 1090 Nakahiro, Ako, Hyogo 678-0232, Japan; Department of Cardiology, Ako City Hospital, 1090 Nakahiro, Ako, Hyogo 678-0232, Japan

**Keywords:** Vasospastic angina, Essential thrombocythemia, Anagrelide, Optical coherence tomography, Histopathology, Case report

## Abstract

**Background:**

Essential thrombocythemia (ET) is a myeloproliferative disorder that increases the thrombotic risk and induces coronary spasms. Anagrelide, a common treatment for ET, has been suspected of trigger coronary spasm potentially leading to acute coronary syndrome (ACS).

**Case summary:**

A 51-year-old woman with ET who was treated with anagrelide developed recurrent ACS caused by refractory vasospastic angina. She initially underwent percutaneous coronary intervention (PCI) with a drug-eluting stent (DES) for severe stenosis of the proximal left anterior descending artery. However, her symptoms recurred, and progressive restenosis at the distal edge of the stent caused by vasospasm was confirmed using optical coherence tomography (OCT), histology, and invasive provocation tests. The patient underwent PCI with DES after directional coronary atherectomy, which helped to reduce the plaque volume and allowed for histopathological analysis. Her ET medication was switched from anagrelide to hydroxyurea and vasodilatory therapy was intensified with benidipine, diltiazem, and nicorandil. Follow-up assessments, including invasive provocation tests under treatment with vasodilators, demonstrated effective suppression of coronary spasm, and the patient remained stable without further ischaemic events.

**Discussion:**

This case underscores the role of coronary spasm in recurrent ACS in patients with ET and highlights the potential side effects of anagrelide. A multidisciplinary diagnostic approach, including OCT and histopathological evaluation and spasm provocation, can help to identify the underlying pathophysiology of ACS. Tailored therapy, verified through invasive provocation tests under vasodilation, successfully controlled the symptoms and prevented recurrence.

Learning points

- Both essential thrombocythemia (ET) and anagrelide, a treatment for ET, can induce coronary spasm.
- A multidisciplinary diagnostic approach, including optical coherence tomography imaging and histopathological evaluation, can be used to identify coronary spasm as the trigger of acute coronary syndrome.
- Assessing residual spasmogenicity using invasive provocation tests under vasodilatation treatments is effective in managing refractory vasospastic angina.

## Introduction

Essential thrombocythemia (ET) is a myeloproliferative disorder caused by the monoclonal proliferation of haematopoietic stem cells. Essential thrombocythemia primarily increases the risk of thrombotic complications, including coronary thrombosis, but may also contribute to the development of coronary spasm.^1,2^ Furthermore, anagrelide, a therapeutic drug used for ET, likely induces coronary spasm as a side effect that can occasionally result in acute coronary syndrome (ACS).^3^

We present the rare case of a middle-aged woman with recurrent ACS caused by refractory vasospastic angina (VSA) while receiving anagrelide for ET. A multidisciplinary diagnostic approach, using optical coherence tomography (OCT), histopathology, and spasm provocation tests, was used to identify the ACS pathophysiology. Invasive provocation tests under vasodilators helped to evaluate treatment efficacy and optimize patient management.

## Summary figure

**Figure:**
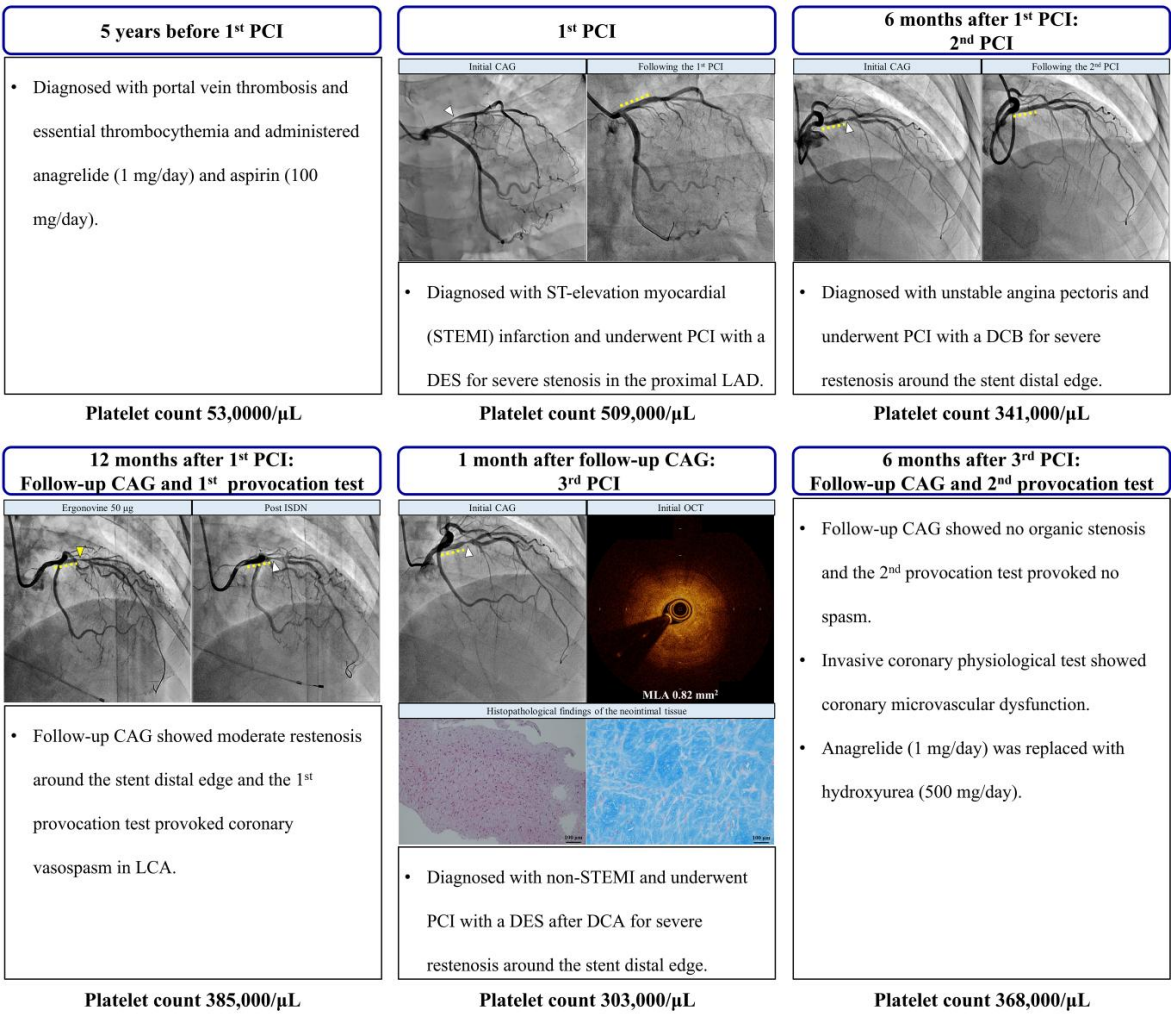
Arrowheads indicate the culprit sites. Dotted lines indicate polymer-free sirolimus-eluting stents in the proximal left anterior descending artery. Yellow arrowheads indicate a diffuse spasm in the middle and distal LAD.

## Case presentation

**Figure 1:**
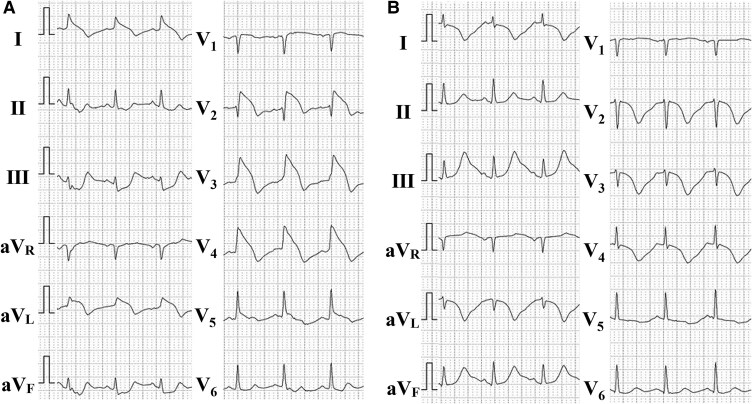
Electrocardiography. Electrocardiogram on admission (*A*) and after isosorbide dinitrate administration (*B*).

**Figure 2:**
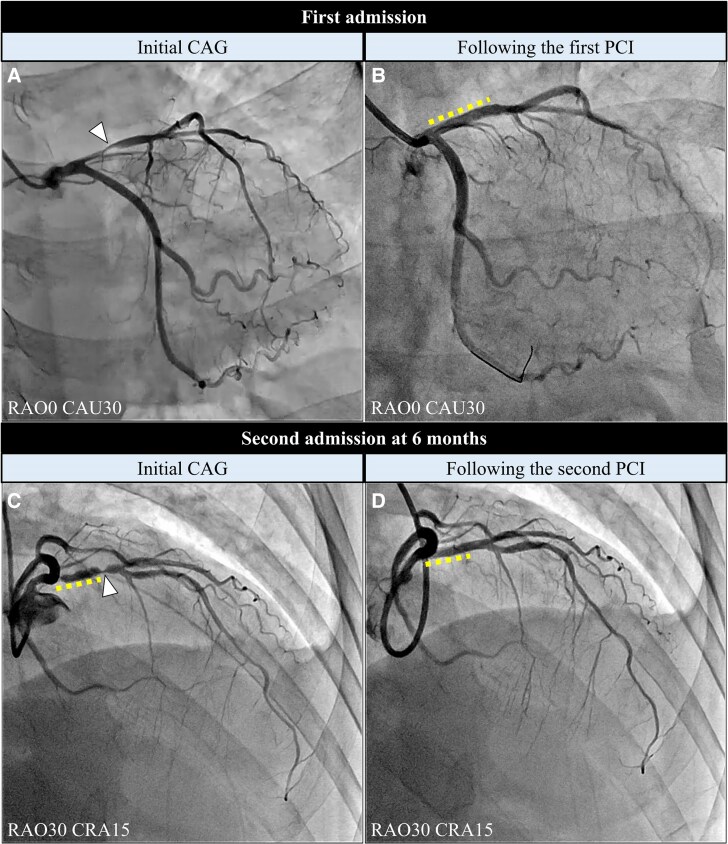
Urgent coronary angiography. Urgent coronary angiography at first admission (*A* and *B*) and second admission at 6 months (*C* and *D*): (*A*) initial coronary angiography, (*B*) following the first percutaneous coronary intervention, (*C*) initial coronary angiography, and (*D*) following the second percutaneous coronary intervention. Arrowheads indicate the culprit sites. Dotted lines indicate polymer-free sirolimus-eluting stents in the proximal left anterior descending artery. CAU, caudal view; CRA, cranial view; RAO, right anterior oblique view.

A 51-year-old female with early morning chest pain was admitted to our hospital. The patient was diagnosed with ET 5 years earlier, followed by portal vein thrombosis, and was treated with anagrelide (1 mg/day) and aspirin (100 mg/day) from the time of diagnosis. She had a history of smoking (15 cigarettes/day for 25 years). Upon admission, her vital signs were as follows: pulse rate, 85 b.p.m.; blood pressure, 155/100 mmHg; and oxygen saturation, 100% in ambient air. Electrocardiography (ECG) indicated sinus rhythm with ST elevation in leads V2–V4, I, and aV_L_, which changed to ST depression in the same leads after the administration of isosorbide dinitrate (ISDN) (*Figure 1*). Initial laboratory tests revealed a platelet count of 509 000/μL. Urgent coronary angiography (CAG) revealed critical stenosis in the proximal left anterior descending (LAD) artery, and *ad hoc* percutaneous coronary intervention (PCI) using a polymer-free sirolimus-eluting stent was performed (*Figure 2*). The patient was discharged with prasugrel (3.75 mg/day), aspirin (100 mg/day), rosuvastatin (10 mg/day), ezetimibe (10 mg/day), and anagrelide (1 mg/day). Six months after the first PCI, the patient was readmitted due to unstable angina. The patient demonstrated good compliance with her prescribed medication, and her platelet count was reduced to 341 000/μL. Urgent CAG revealed severe stenosis distal to the stent edge in the proximal LAD segment. The second PCI was performed using a drug-coated balloon (*Figure 2*). After discharge, benidipine (8 mg/day) was added for suspected VSA, as residual attacks continued.

**Figure 3:**
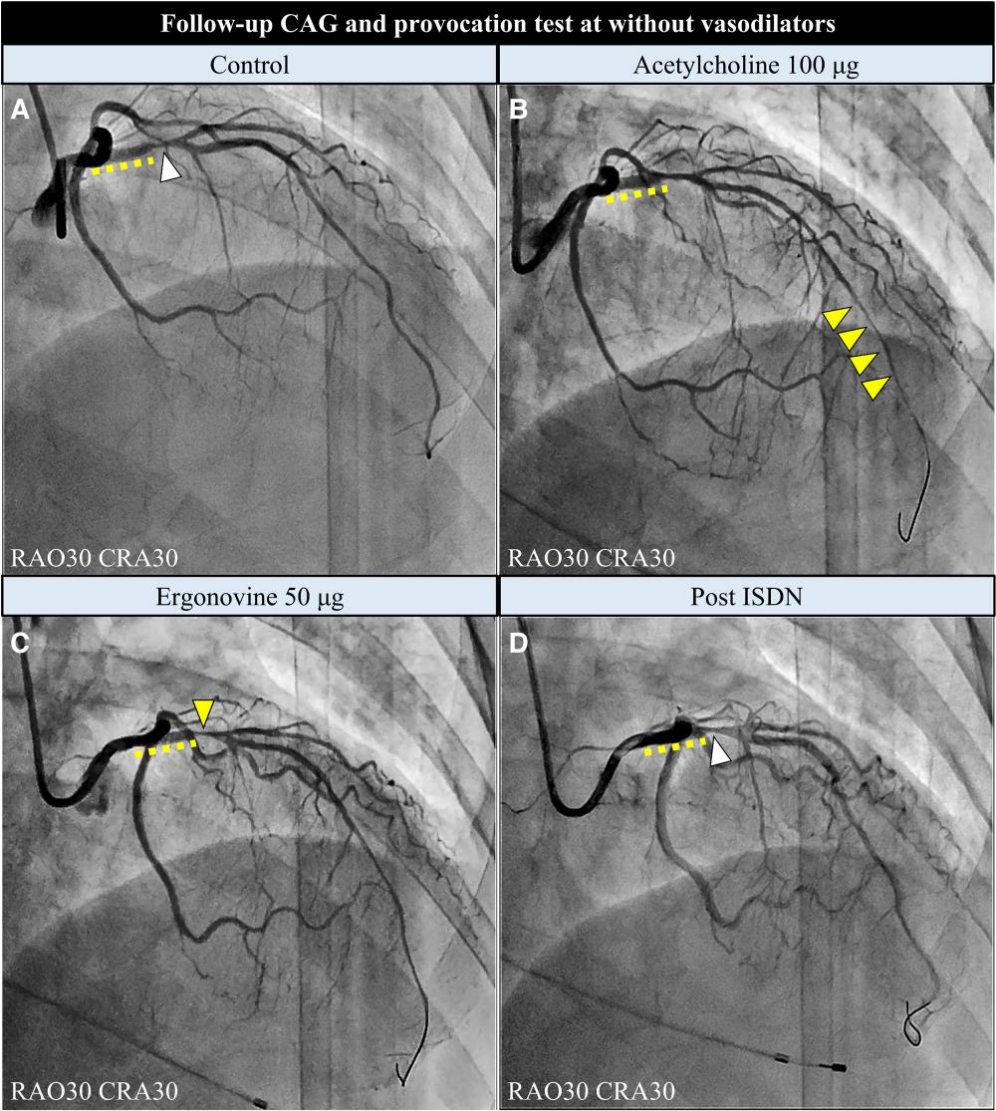
Follow-up coronary angiography and spasm provocation tests without vasodilators. (*A*) Follow-up coronary angiography image showing moderate restenosis (arrowheads) distal to the stent. (*B*) Coronary angiography provoked by acetylcholine 100 μg, showing a diffuse spasm in the middle and distal LAD (arrowheads). (*C*) Coronary angiography after intracoronary ergonovine, showing focal spasm in the proximal left anterior descending artery, consistent with the restenosis site (arrowheads). (*D*) Post-intracoronary injection of isosorbide dinitrate. Dotted lines indicate a polymer-free sirolimus-eluting stent in the proximal left anterior descending artery. LAD, left anterior descending artery.

**Figure 4:**
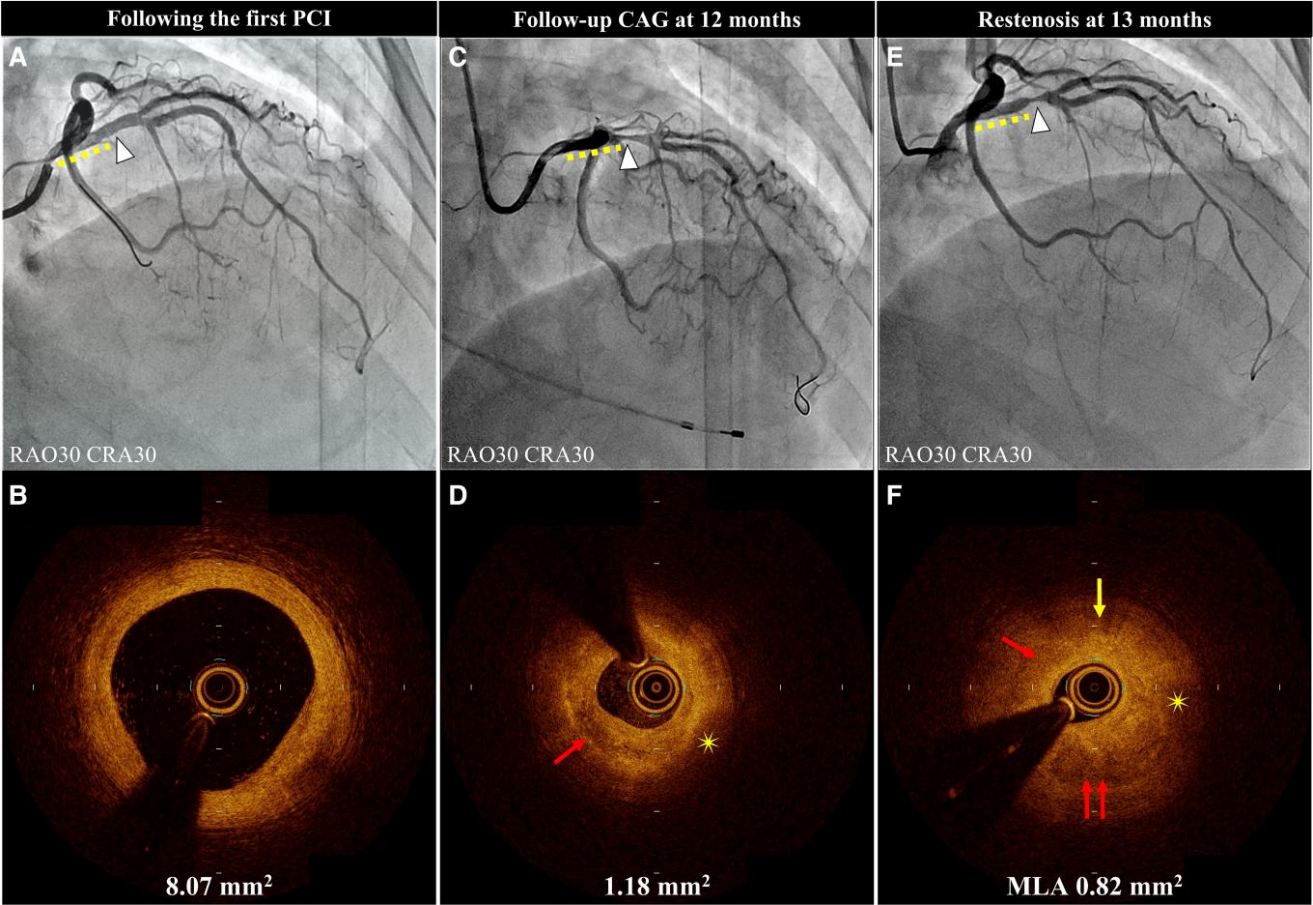
Serial coronary angiography and optical coherence tomography images. Coronary angiography and optical coherence tomography images after the first percutaneous coronary intervention (*A* and *B*), at 12-months follow-up (*C* and *D*), and at restenosis (13 months) (*E* and *F*). The arrowhead indicates the restenosis site. Yellow dotted lines indicate polymer-free sirolimus-eluting stents in the proximal left anterior descending artery. Optical coherence tomography showing a layered plaque (arrows), an intraplaque haemorrhage (asterisk), and cholesterol crystals (arrow). MLA, minimum lumen area.

One year after the first PCI, follow-up CAG and the first spasm provocation tests were performed after withdrawal of benidipine. The platelet count was well controlled at 385 000/μL with anagrelide (1 mg/day). The CAG revealed a moderate restenosis of the LAD artery around the distal edge of the stent. Acetylcholine injection (100 μg) into the left coronary artery caused transient 90% stenosis, indicating diffuse spasms in the middle and distal LAD arteries accompanied by chest pain and ST depression in leads V1–V6. Ergonovine (50 μg) provoked 90% focal spasm in the proximal LAD consistent with restenosis site distal to the stent (*Figure 3*). Optical coherence tomography revealed a reduction in the minimum lumen area (MLA) from 1.18 to 0.67 mm^2^ at the restenosis lesion after ergonovine injection, suggesting stenosis progression probably due to coronary spasm, and layered plaques at this lesion site (*Figure 4*). After intracoronary administration of ISDN, the coronary spasm resolved and the ECG findings normalized. The fractional flow reserve of the LAD artery yielded a value of 0.86; therefore, revascularization was deferred. Consequently, benidipine was resumed and isosorbide mononitrate (40 mg/day) was added.

**Figure 5:**
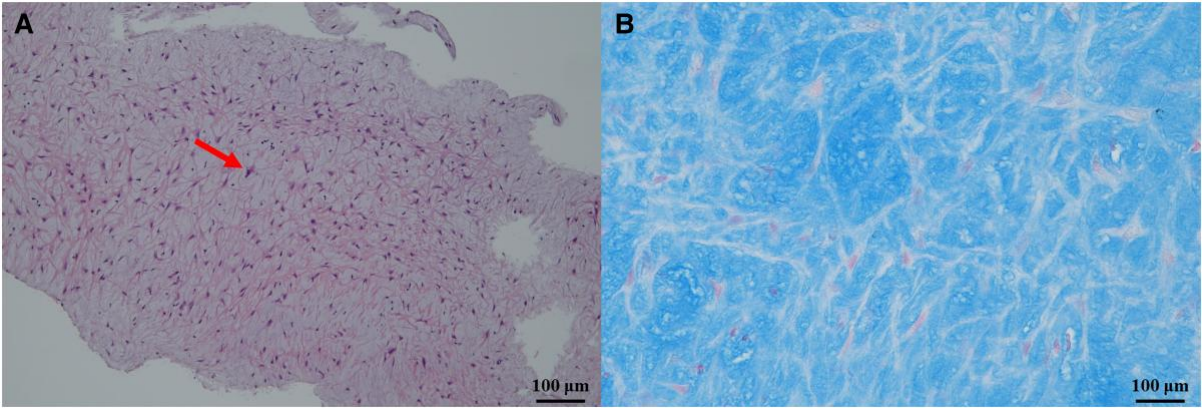
Histopathological findings from endomyocardial biopsy. Histopathological findings of the neointimal tissue obtained by directional coronary atherectomy revealed stellate-shaped smooth muscle cells (arrow) within the acidic mucopolysaccharide-rich myxedematous stroma, suggesting neointimal hyperplasia. Haematoxylin and eosin (*A*) and Alcian blue (*B*) staining were performed.

One month after the follow-up CAG, the patient was readmitted with chest pain refractory to oral vasodilators. Electrocardiography revealed ST elevation in leads V1–V4, resolved with nitroglycerin. The platelet level was stable at 303 000/μL. Urgent CAG demonstrated the progression of residual stenosis in the LAD artery compared to 1 month earlier. Optical coherence tomography showed layered plaques, intraplaque haemorrhage, and cholesterol crystals without thrombus or spontaneous coronary dissection, indicating plaque instability probably caused by repeated vasospasms and a reduced MLA of 0.82 mm^2^ in the culprit lesion. Directional coronary atherectomy was performed to reduce the plaque volume and investigate the histology of the refractory neointimal progression, followed by implantation of a durable polymer everolimus-eluting stent (*Figure 4*). Histopathology revealed smooth muscle cells within Alcian blue–positive myxoedematous stroma, suggesting neointimal hyperplasia (*Figure 5*). Optical coherence tomography and histopathology revealed rapid plaque progression probably due to coronary spasm. The patient was discharged after receiving an additional course of diltiazem (100 mg/day) and reported occasional chest pain after discharge. Nicorandil (15 mg/day) was added and the diltiazem dose was increased to 200 mg/day, which relieved her symptoms.

Six months after the third PCI, a follow-up CAG and a second provocation test under vasodilators (benidipine, 8 mg/day; diltiazem, 200 mg/day; isosorbide mononitrate, 40 mg/day; nicorandil, 15 mg/day) and an invasive coronary physiological test with a pressure wire (PressureWire™ X, Abbott, USA) were performed. This examination revealed no organic stenosis or spasms in the coronary arteries, coronary flow reserve (CFR) of 1.8, the index of microcirculatory resistance (IMR) of 31 U, and a resistive reserve ratio of 2.3, meeting the relevant diagnostic criteria for coronary microvascular dysfunction (CMD) (CFR < 2.0, IMR ≧ 25). After discharge, anagrelide (1 mg/day) was replaced with hydroxyurea (500 mg/day), which is a potential causative agent of refractory VSA. We conclude that this treatment effectively suppressed the patient’s spasmogenicity.

## Discussion

Here, we report a rare case of recurrent ACS and refractory VSA in a patient receiving anagrelide for ET. A multidisciplinary diagnostic approach using histology, OCT assessment, and invasive provocation tests demonstrated that coronary spasm was the trigger for recurrent ACS. In ET, the release of platelet-derived vasospasm-promoting substances, such as serotonin and thromboxane A2, induces coronary spasms and may contribute to ACS.^1^ Conversely, anagrelide, a key drug in ET treatment, can cause coronary spasm-related ACS, which is rarely reported in patients (1%–5%).^3^ Anagrelide is a type III cyclic adenosine monophosphate-phosphodiesterase inhibitor. In animal models, phosphodiesterase inhibitors have been shown to increase transmitter release from sympathetic nerves, hypothesized to underlie their side effects on human epicardial coronary arteries.^3,4^ The patient had no chest pain before anagrelide administration and her platelet count was well controlled, suggesting that this event was caused by anagrelide rather than by ET itself; therefore, anagrelide was replaced with hydroxyurea.

Previous studies reported histopathological findings of spastic lesions in patients with VSA. Histopathological evaluation of coronary plaques at spastic sites demonstrates neointimal hyperplasia consisting of smooth muscle cells with a myxomatous extracellular matrix, thrombus formation, and intimal haemorrhage more frequently than in chronic stable angina.^5,6^ Neointimal hyperplasia is a rapid vascular remodelling response to injury, often following intimal haemorrhage and mural thrombus triggered by coronary spasms at spastic sites. They play a key role in the rapid progression of organic stenosis at these sites.^5^

In addition to histopathological considerations, OCT can be used to visualise progressive lesions and vessel injury at provoked spastic sites in patients with VSA. Optical coherence tomography images frequently show layered plaques and inflammatory characteristics, such as macrophage infiltration and intraplaque microchannels in organic stenosis at provoked spastic sites. Layered plaques suggest a healing process in which a silent thrombus is replaced by proteoglycan-rich granulation tissue.^7^ Coronary spasms have been reported to induce local thrombus formation and active inflammatory responses, thereby increasing the risk of rapid plaque progression and ischaemic events in patients.^7,8^

Based on the histopathological and OCT findings, the present case was likely a case of spasm-induced ACS with no thrombus or intimal tear in the culprit lesion and only layered plaques. Additionally, we performed provocation tests using vasodilators and confirmed the suppression of spasmogenicity using a multidrug combination. Provocation tests under vasodilators have been reported to be useful in confirming drug efficacy and determining the optimal medication for VSA.^9^

Patients diagnosed with VSA frequently demonstrate generalized vascular dysfunction, extending beyond focal coronary spasm to include the coronary microcirculation,^10^ and CMD has been associated with an elevated risk of major adverse cardiovascular events.^11^ In this case, the coexistence of VSA and CMD may have contributed to the recurrence of ACS.

## Conclusion

We report a rare case of recurrent ACS caused by refractory VSA, possibly due to anagrelide, which was confirmed using OCT and physiological and histopathological evaluations. Provocation tests under vasodilators verified the suppressed spasmogenicity in refractory VSA. Since refractory coronary spasms can rapidly exacerbate organic stenosis, the suppression of spasmogenicity is crucial.

## Lead author biography


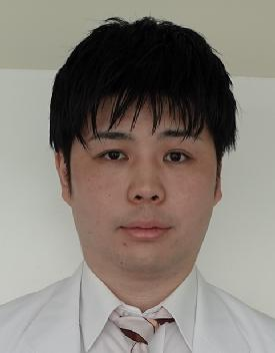


Fujii Masayoshi graduated from Hyogo college of Medicine in 2016. He completed residency programme at Steel Memorial Hirohata Hospital and worked as a fellow in cardiology at Division of Cardiovascular Medicine, Department of Internal Medicine, Kobe University Graduate School of Medicine, Division of Cardiovascular Medicine, Hyogo Brain and Heart Center, Himeji, Japan.

## Data Availability

The data underlying this article are available from the corresponding author upon reasonable request. De-identified supporting files can be shared.

## Notes

**Funding:** None declared.
